# Ultrasound-derived fat fraction for detection of hepatic steatosis and quantification of liver fat content

**DOI:** 10.1007/s11547-023-01693-8

**Published:** 2023-08-12

**Authors:** Riccardo De Robertis, Flavio Spoto, Daniele Autelitano, Daniela Guagenti, Antonia Olivieri, Piero Zanutto, Greta Incarbone, Mirko D’Onofrio

**Affiliations:** 1 Department of Radiology, Ospedale G.B. Rossi AOUI Verona, 37134 Verona, Italy; 2 Department of Diagnostics and Public Health, University of Verona, Piazzale L.A. Scuro 10, 37134 Verona, Italy

**Keywords:** Liver, Fatty liver, Non-alcoholic fatty liver disease, Ultrasound, Magnetic resonance

## Abstract

**Purpose:**

To compare ultrasound (US) and US-derived fat fraction (UDFF) with magnetic resonance proton density fat fraction (MRI-PDFF) for the detection of hepatic steatosis and quantification of liver fat content.

**Materials and methods:**

Between October and December 2022, 149 patients scheduled for an abdominal MRI agreed to participate in this study and underwent MRI-PDFF, US and UDFF. Inclusion criteria were: (a) no chronic liver disease or jaundice; (b) no MRI motion artifacts; (c) adequate liver examination at US. Exclusion criteria were: (a) alcohol abuse, chronic hepatitis, cirrhosis, or jaundice; (b) MRI artifacts or insufficient US examination. The median of 10 MRI-PDFF and UDFF measurements in the right hepatic lobe was analyzed. UDFF and MRI-PDFF were compared by Bland–Altman difference plot and Pearson’s test. Sensitivity, specificity, positive and negative predictive values, accuracy, and area under the receiver-operator curve (AUC-ROC) of US and UDFF were calculated using an MRI-PDFF cut-off value of 5%. *p* values ≤ 0.05 were statistically significant.

**Results:**

122 patients were included (61 men, mean age 60 years, standard deviation 15 years). The median MRI-PDFF value was 4.1% (interquartile range 2.9–6); 37.7% patients had a median MRI-PDFF value ≥ 5%. UDFF and MRI-PDFF had high agreement (*p* = 0.11) and positive correlation (*⍴* = 0.81, *p* < 0.001). UDFF had a higher diagnostic value than US for the detection of steatosis, with AUC-ROCs of 0.75 (95% CI 0.65, 0.84) and 0.53 (95% CI 0.42, 0.64), respectively.

**Conclusions:**

UDFF reliably quantifies liver fat content and improves the diagnostic value of US for the detection of hepatic steatosis.

## Introduction

Non-alcoholic fatty liver disease (NAFLD) is the most common liver disease, with an estimated prevalence of 5–30%, resulting in more than 1 billion people affected worldwide [1, 2]. The histopathologic spectrum of NAFLD ranges from intracellular lipid accumulation to coexisting inflammation, hepatocyte swelling, and fibrosis; the disease can progress to cirrhosis and its complications, including hepatocellular carcinoma (HCC) [3]. It is important to diagnose NAFLD at an early stage, when disease evolution is potentially reversible through healthy lifestyle measures that have been shown to reduce histological fat deposition in the liver [4]. The diagnosis of NAFLD is based on three criteria: absence of alcohol abuse, presence of histologically documented fat deposition greater than 5%, and exclusion of other liver diseases [5]. The gold standard for diagnosing and staging NAFLD is liver biopsy, although this method is invasive, relatively expensive, and does not allow the longitudinal evaluation of the same patient to monitor the impact of any therapeutic intervention as well as disease evolution [6, 7]. Transabdominal ultrasound (US) is the most commonly used imaging method in subjects with suspected NAFLD, as it is inexpensive, widely available, and repeatable. Nevertheless, US is limited in obese and uncooperative patients, has a low sensitivity for the diagnosis of mild steatosis, and lacks reproducibility and objectivity. Measurement of hepatic fat content using magnetic resonance imaging (MRI) overcomes these limitations, and the most consistent and reproducible results were obtained using MRI proton density fat fraction (MRI-PDFF), which is considered the gold standard for the noninvasive quantification of liver fat as it demonstrated significant correlation with histologic steatosis grade and reproducibility in previous studies [8, 9]. Nonetheless, MRI-PDFF has a limited clinical application owing to cost and availability and cannot be routinely used for screening and longitudinal surveillance. In this view, quantitative US techniques have been developed to overcome the limitations of B-mode US and improve its diagnostic capabilities to detect and quantify hepatic steatosis rapidly and with high reliability and reproducibility. Ultrasound-derived fat fraction (UDFF) is a commercially available quantitative US-based method that can estimate liver fat content from both the attenuation coefficient (AC) and the backscatter coefficient (BSC) without the need to acquire reference phantom scans after each liver scan by having reference phantom data integrated into the US system [10]. Quantification of liver fat content with UDFF is directly and rapidly acquired by placing a ROI in the liver parenchyma [10]. A study performed on overweight and obese patients demonstrated strong agreement between UDFF and MRI-PDFF measurements [11]. Although UDFF may therefore suit well as a screening tool for hepatic steatosis, very few data are available regarding its use in clinical practice [12].

The aim of this study was to prospectively compare B-mode US and UDFF with MRI-PDFF for detection of hepatic steatosis and quantification of liver fat content.

## Materials and methods

The IRB waived the need for patient informed consent, as the results of this research would not affect their clinical care.

### Study population

**Fig. 1:**
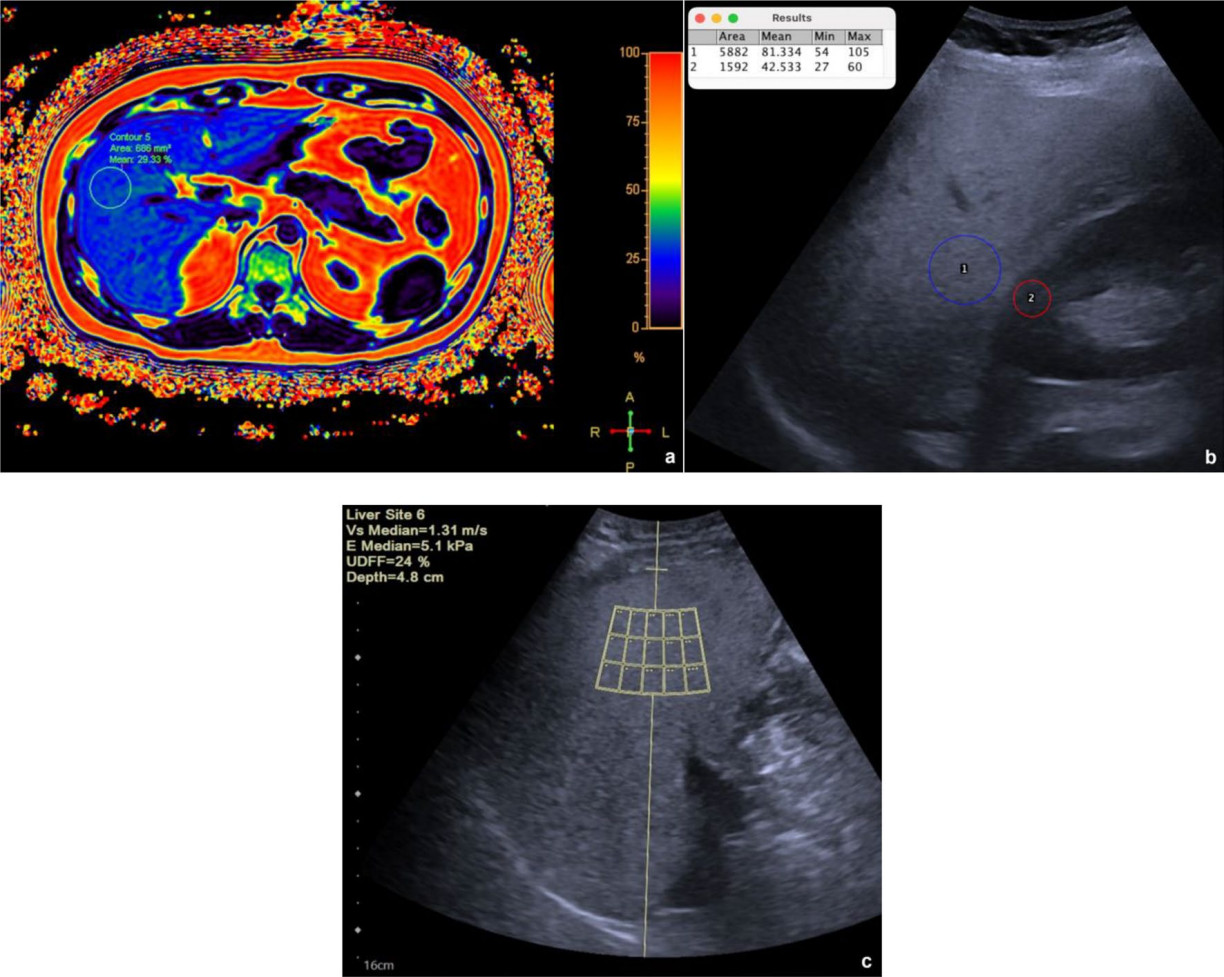
Quantification of liver fat content in a 31-year-old man with NAFLD. **a** Fat fraction map: a circular ROI is visible in liver segment 5; the overall median MRI-PDFF value was 25%. **b** B-mode US and quantification of the hepatorenal index: liver echogenicity is higher than the right kidney cortex; visualization of the diaphragm and posterior surface of the right hepatic lobe is decreased; the ratio of the mean brightness of liver (1) and kidney (2) ROIs was 1.91; **c** UDFF measurement: a ROI box is visible in liver segment 5; the overall median UDFF value was 24%

**Fig. 2:**
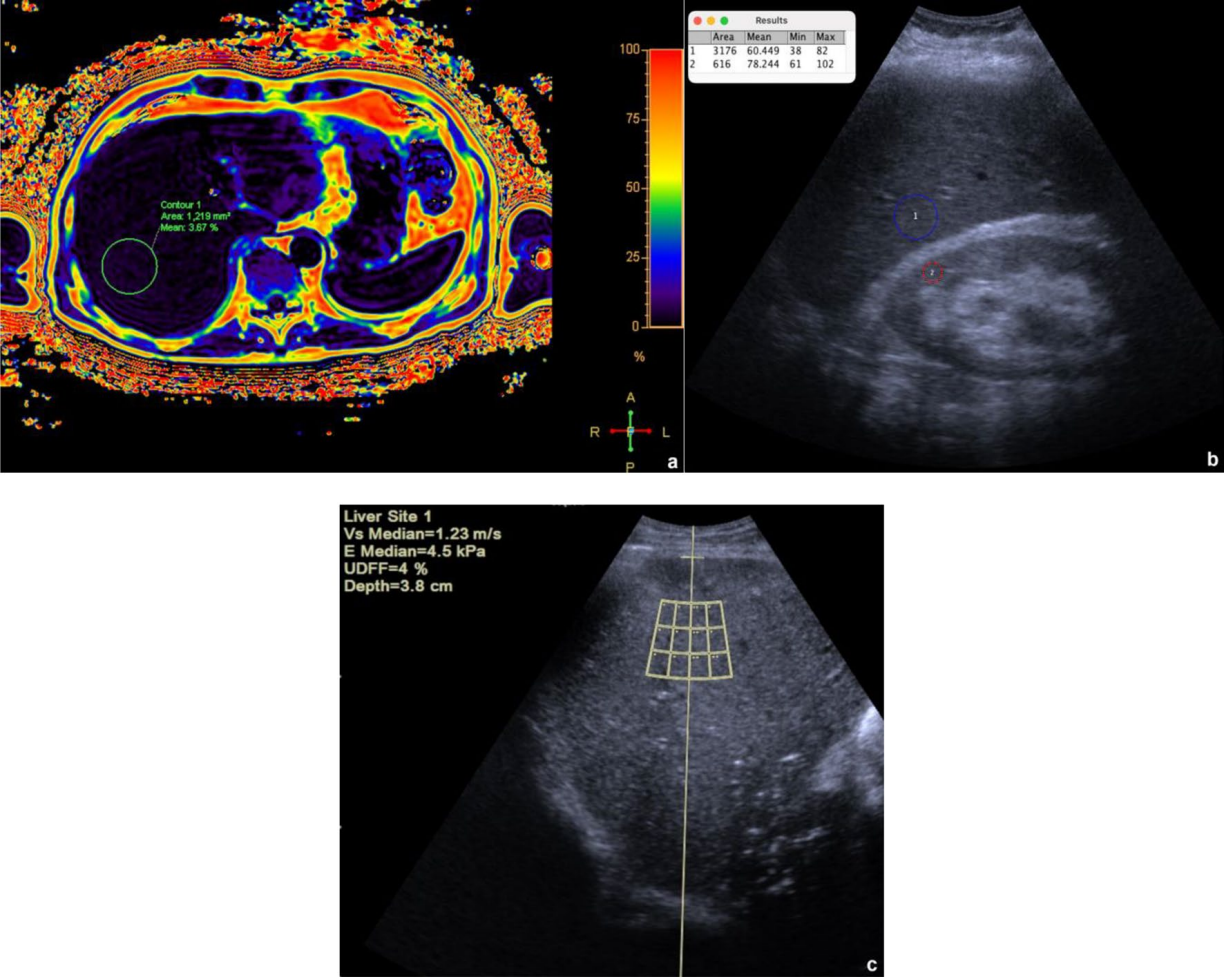
Quantification of liver fat content in a 58-year-old woman without steatosis. **a** Fat fraction map: a circular ROI is visible in liver segment 7; the overall median MRI-PDFF value was 4%. **b** B-mode US and quantification of the hepatorenal index: liver echogenicity is comparable to the right kidney cortex; the ratio of the mean brightness of liver (1) and kidney (2) ROIs was 0.77; **c** UDFF measurement: a ROI box is visible in liver segment 7; the overall median UDFF value was 4%

Patients scheduled for an abdominal MRI examination between October and December 2022 were offered to participate in this prospective study; 149 of them agreed and underwent MRI-PDFF and transabdominal US, including B-mode evaluation and UDFF on the same day (Figs. 1, 2). Patients had fasted for 6 h before examinations. Inclusion criteria were: (a) absence of chronic liver disease and/or jaundice; (b) absence of motion artifacts on MRI images; (c) adequate US examination of the liver. Exclusion criteria were: (a) history of alcohol abuse, presence of chronic viral hepatitis, hemochromatosis, cirrhosis, or jaundice; (b) uncooperative patients with presence of major artifacts on MRI images and/or inadequate US explorability of the liver. Patients’ body mass index (BMI) was calculated as body weight divided by the square of height (kg/m^2^). Aspartate and alanine aminotransferase (AST and ALT), gamma-glutamyl transferase (GGT), and bilirubin levels were retrieved from the most recent blood test.

### MRI-PDFF measurements

MRI-PDFF was acquired on a 3T scanner (Ingenia Elition S; Philips Healthcare). Examinations were performed with patients in supine position using a torso phased-array coil. MRI-PDFF was obtained with the mDIXON Quant pulse sequence, using a 6-echo acquisition during a single breath hold. The technical parameters of the sequence were as follows: imaging plane, axial; time of repetition (TR)/first time of echo (TE)/delta TE, 6.1/1.08/0.8 ms; field of view, 400 × 325 mm; acquisition matrix, 168 × 129; reconstruction matrix, 640 × 640; flip angle, 3°; section thickness, 6 mm; acquisition voxel, 2.4 × 2.5 × 6 mm; reconstruction voxel, 0.6 × 0.6 × 3 mm. This sequence provided a three-dimensional fat fraction (FF) map covering the whole liver. A radiology resident placed 10 regions of interest (ROIs) on the FF map on different levels in the right hepatic lobe, under the supervision of a senior radiologist (M.D.O.); ROIs were as large as possible, excluding vessels, bile ducts, and liver lesions. The median of these measurements was calculated and used for analysis.

### US examination and measurements

US examinations were performed with a commercially available equipment (ACUSON Sequoia US system, version VA40; Siemens Medical Solution) using a 9C2 convex probe. US examinations were performed by a radiologist with 10 years of experience (R.D.R.) blinded to MRI-PDFF results. B-mode US imaging was first performed to ensure the patient’s cooperativeness and explorability of the whole liver; liver appearance was recorded. B-mode images were transferred to a personal computer to calculate the hepatorenal index by comparing the brightness of two ROIs placed in liver segment 6 and in the right kidney cortex using the software ImageJ [13]. The ROIs were as large as possible, avoiding major vessels, ducts, and lesions. Patients were categorized as having or not hepatic steatosis by considering both the US findings indicative of hepatic steatosis, such as increased echogenicity of the liver parenchyma in comparison to the right kidney cortex and reduced visualization of the diaphragm, intrahepatic vessel borders, and posterior surface of the right hepatic lobe [14], and a hepatorenal index value greater than 1.28 [15]. The same operator performed UDFF measurements by placing a 3 × 3 cm ROI on different levels in the right hepatic lobe. The horizontal line appearing with the ROI marker was aligned to the Glisson's capsule to maintain the appropriate 1.5 cm depth from the liver capsule to the top of the box; major intrahepatic vessels, bile ducts, or liver lesions were excluded from the measurement. Measurements were acquired 10 times for each patient, and the median of the measurements was used for analysis.

### Statistical analysis

The cut-off value for the diagnosis of hepatic steatosis was 5% both for MRI-PDFF and UDFF, as determined by previous studies [16, 17]. Agreement and correlation between UDFF and MRI-PDFF were evaluated by the Bland–Altman difference plot, one-sample T test, and Pearson’s correlation test. The diagnostic value of B-mode US and UDFF measurements for the detection of steatosis was assessed by the calculation of sensitivity, specificity, positive and negative predictive values (PPV and NPV), and accuracy. Receiver operating characteristic curves (ROCs) were constructed for B-mode US and UDFF by taking MRI-PDFF as the standard of reference, and area under the curves (AUC-ROC) were calculated; comparison of ROC curves was performed with the method described by Delong et al. [18]. *p* values ≤ 0.05 were considered statistically significant. Statistical analysis was performed using commercially available software (SPSS 23, IBM; and MedCalc 17.9, MedCalc Software).

## Results

### Study population

Twenty-seven patients were excluded from this study owing to the following causes: hemochromatosis (*N* = 11), jaundice (*N* = 5), chronic viral hepatitis (*N* = 3), inadequate US explorability of the liver (*N* = 3), history of alcohol abuse (*N* = 2), major motion artifacts on MRI images (*N* = 2), and cirrhosis secondary to autoimmune hepatitis (*N* = 1). The final cohort of this study included 122 patients, 61 males (50%) and 61 females (50%), with a median age of 62 years (interquartile range—IQR, 52–72 years). Demographic and clinical characteristics of the study cohort are reported in Tables 1 and 2. The median BMI was 24.2 (IQR 21.8–27). The median AST, ALT, GGT, and bilirubin levels were 25 (IQR 20–34) U/L, 20 (18–37) U/L, 20 (14–33) U/L, and 0.6 (IQR 0.4–1) mg/dL, respectively; overall, 19/122 patients (15.6%) had increased transaminase levels. The median MRI-PDFF value in the cohort was 4.1% (IQR 3–6%), with 46/122 patients (37.7%) having a median MRI-PDFF value ≥ 5%, consistent with the presence of liver steatosis.

**Table 1:** Qualitative characteristics of the study population

Variable	Number of cases (%)
Number of patients	122 (100)
Sex	
Men	61 (50)
Women	61 (50)
Increased transaminase levels	
No	103 (84.4)
Yes	19 (15.6)
Steatosis	
No	76 (62.3)
Yes	46 (37.7)

Data are expressed as number of cases and relative percentages

*M* male, *F* female, *N* no, *Y* yes

**Table 2:** Quantitative characteristics of the study population

Variable	Median (IQR)
Age (years)	62 (52–72)
BMI	24.2 (21.8–27)
AST (U/L)	25 (20–34)
ALT (U/L)	20 (18–37)
GGT (U/L)	20 (14–33)
Totale bilirubin (mg/dL)	0.6 (0.4–1)
PDFF (%)	4.1 (3–6)

Data are expressed as median values (interquartile range)

*IQR* interquartile range, *BMI* body mass index, *AST* aspartate aminotransferase, *ALT* alanine aminotransferase, *GGT* gamma-glutamyl transferase, *PDFF* proton density fat fraction

### Agreement and correlation between UDFF and MRI-PDFF

**Fig. 3:**
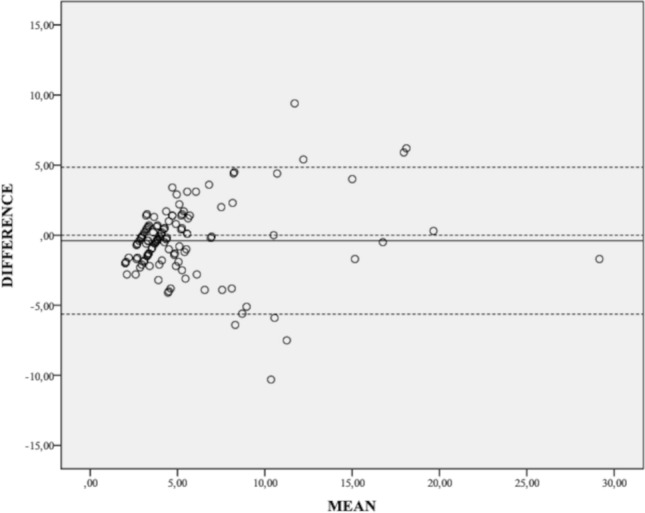
Results of Bland–Altman analysis. The mean difference in liver fat content measured by PDFF and UDFF is 0.35 (solid line). The upper and lower 95% limits of agreement were 4.85 and − 5.63, respectively (dashed lines)

**Fig. 4:**
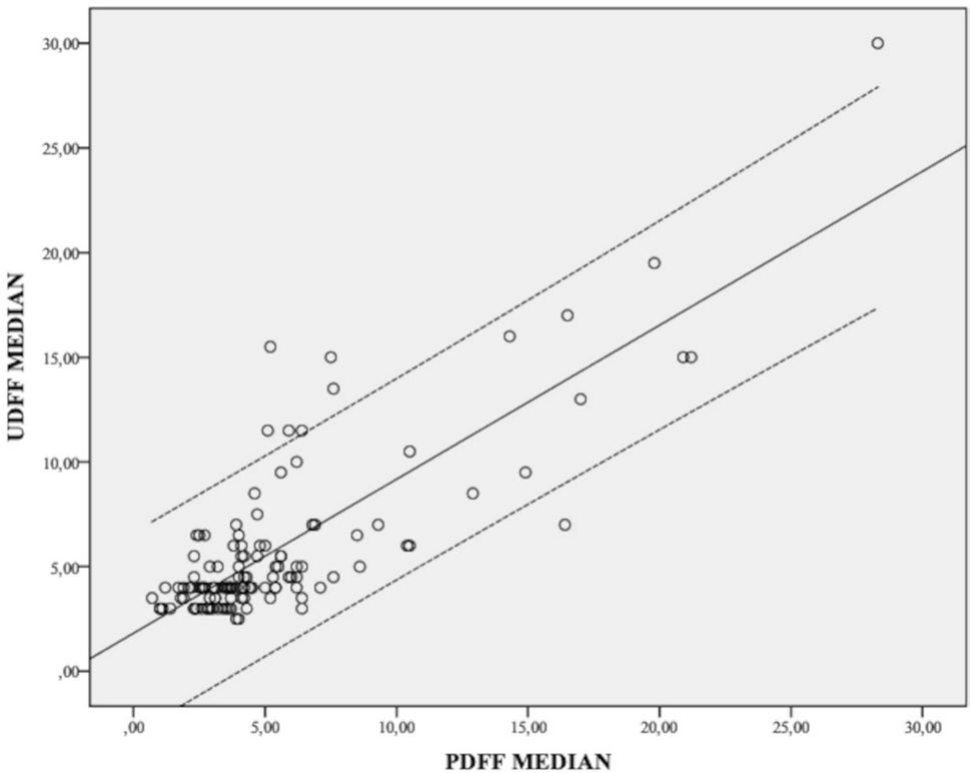
Scatter plot showing the correlation in liver fat content (%) values measured between UDFF and PDFF. The linear regression indicates a strong correlation between the two methods (*⍴* = 0.808, *p* < 0.001). The dashed lines are the 95% limits of agreement

The Bland–Altman analysis showed high agreement between UDFF and MRI-PDFF measurements (Fig. 3), with a mean difference between the mean value of liver fat percentage measured by MRI-PDFF and UDFF of − 0.39. One sample *T*-test showed a nonsignificant difference between the two methods of measurement (*p* = 0.110). The Pearson’s test revealed a significant, positive, and strong correlation between the two methods (*⍴* = 0.808, *p* < 0.001; Fig. 4).

### Detection of hepatic steatosis

**Fig. 5:**
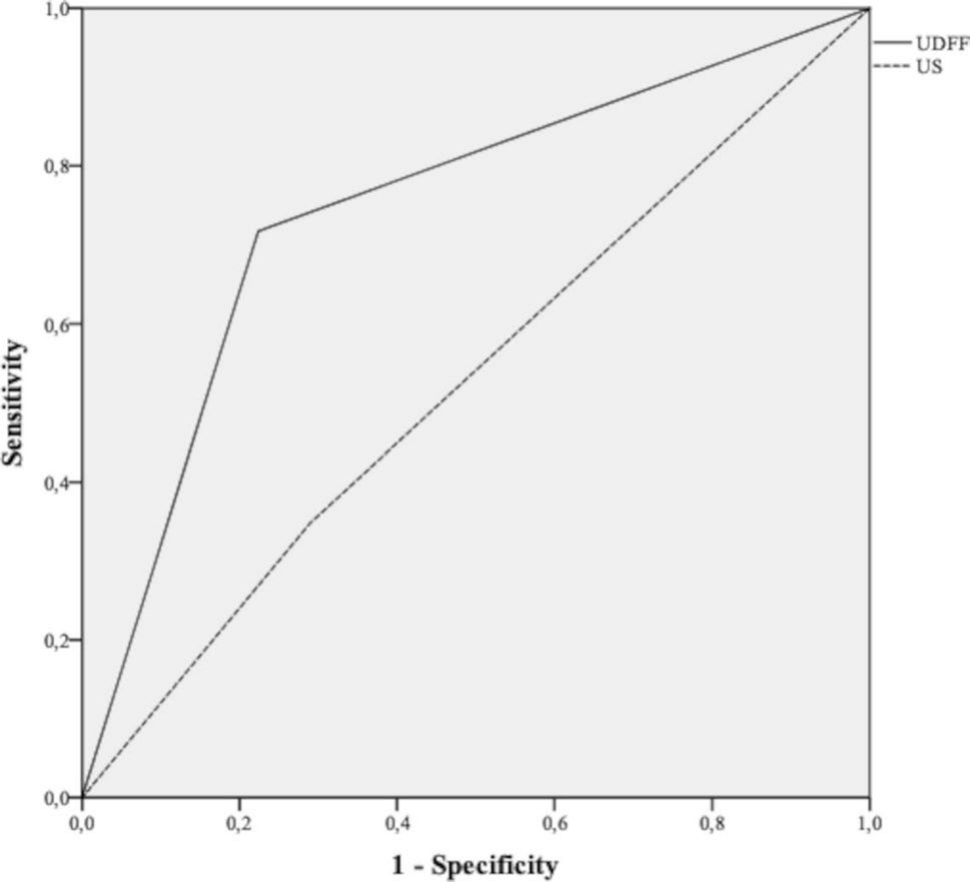
AUC-ROC curves of UDFF (solid line) and B-mode US (dashed line) for detection of steatosis

The diagnostic value of B-mode US and UDFF for the detection of hepatic steatosis is reported in Tables 3 and 4. ROC curves are presented in Fig. 5. B-mode US had sensitivity, specificity, PPV, NPV, and accuracy values for detection of liver steatosis of 34.8%, 71.1%, 42.1%, 64.3%, and 57.5%, respectively. UDFF performed better than B-mode US, with sensitivity, specificity, PPV, NPV, and accuracy values for detection of liver steatosis of 71.1%, 77.6%, 66%, 82%, and 75.4%, respectively. The AUC-ROC of B-mode US and UDFF was 0.529 and 0.747, respectively; the comparison of ROC curves showed a statistically significant difference (*p* = 0.03; Fig. 5).

**Table 3:** Diagnostic value of B-mode US for detection of steatosis

Statistic	Value (%)	95% CI
Sensitivity	34.8	21.4–50.3
Specificity	71.1	59.5–80.9
PPV	42.1	26.3–59.2
NPV	64.3	53.1–74.5
Accuracy	57.4	48.1–66.3
AUC-ROC	0.53	0.42–0.64

Data are expressed as %

*95% CI* 95% confidence interval, *PPV* positive predictive value, *NPV* negative predictive value, *AUC-ROC* area under the receiver operator curve

**Table 4:** Diagnostic value of UDFF for detection of steatosis

Statistic	Value (%)	95% CI
Sensitivity	71.7	56.5–84
Specificity	77.6	66.6–86.4
PPV	66	51.2–78.8
NPV	82	71.1–90
Accuracy	75.4	66.8–82.8
AUC-ROC	0.75	0.65–0.84

Data are expressed as %

*95% CI* 95% confidence interval, *PPV* positive predictive value, *NPV* negative predictive value

## Discussion

This prospective study evaluated the clinical application of UDFF for the detection of hepatic steatosis and quantification of hepatic fat content. The standard of reference was MRI-PDFF, which is considered the standard of reference for non-invasive test for liver fat [8], as it demonstrated an excellent correlation with histologically graded steatosis as well as high repeatability and reproducibility [9]. We found a strong agreement between UDFF and MRI-PDFF measurements, with a minimal, nonsignificant difference between the mean value of liver fat percentage measured by the two methods at Bland–Altman analysis and one-sample T test. Moreover, the measurements obtained by the two methods had a significant, positive, and strong correlation at Pearson’s test (*⍴* = 0.808, *p* < 0.001). These results highlight the reliability of UDFF in the quantification of liver fat content. In our study, B-mode US had very low sensitivity (34.8%), PPV (42.1%), and accuracy (57.5%) for the detection of hepatic steatosis, while UDFF performed significantly better, with sensitivity of 71.1%, specificity of 77.6%, PPV of 66%, NPV of 82%, and accuracy of 75.4%. AUC-ROCs of B-mode US and UDFF were 0.529 and 0.747, respectively, with a significant difference between the ROC curves of the two methods (*p* = 0.03). In light of that, a quantitative approach to liver US based on UDFF measurement may allow for a higher detection rate of patients with hepatic steatosis, even at an early stage of disease. Early diagnosis of NAFLD is important, as disease evolution is potentially reversible through healthy lifestyle measures. Transabdominal US should be used as the primary imaging method to identify hepatic steatosis because it is widely available and cheap [1]. Nevertheless, B-mode US has a limited sensitivity and does not reliably detect steatosis when lower than 20% [14, 19]. The diagnostic gap in these patients may be filled using quantitative measurement methods implemented in US equipments, that estimate the AC, a measure of the loss of US energy in tissues, and the BSC, which is related to tissue microstructure [10, 20]. Due to the novelty of this method, limited study data are available concerning the diagnostic value of UDFF in detecting hepatic steatosis in a clinical setting. Gao et al. [12] prospectively evaluated 21 subjects and reported high intra-observer repeatability and inter-observer reproducibility for UDFF measurements (> 0.85); UDFF demonstrated a high correlation with MRI-PDFF results. Dillman et al. [11] reported a mean bias between UDFF and MRI-PDFF of 4.0%, with a significant, positive association between the two methods (*ρ* = 0.82; *p* < 0.001). Labyed et al. [17] reported a Pearson’s correlation coefficient between UDFF and MRI-PDFF of 0.87. However, these studies were conducted on overweight and obese patients and subjects with known or suspected NAFLD, respectively; instead, our results were based on a prospective, mixed cohort of subjects that is representative of the general European population. Beside the potential role of UDFF to complement or replace MRI-PDFF for the longitudinal evaluation of NAFLD patients enrolled in clinical trials, our results support the clinical role of UDFF in the quantification of liver fat content in the general population.

Even though the sample size was substantial and reflected demographic and clinical variety, our study has several limitations. First, owing to the prospective nature of this study, our cohort was mainly composed of healthy subjects, while all positive subjects had mild steatosis; consequently, while on the one hand our detection performance was lower than that of previous studies [11, 17], on the other hand we did not test the diagnostic performance of UDFF in patients with steatosis grade ≥ 2. Second, none of the patients underwent liver biopsy. While the reliability of MRI-PDFF in determining the liver fat content has been established by previous studies [8, 9], we did not explore the potential impact of coexisting inflammation or fibrosis on UDFF measurements. Third, although multiple UDFF measurements were performed, examinations were performed by a single radiologist, and this may have resulted in underestimating measurement variability.

In conclusion, quantification of liver fat content using UDFF is reliable and has clinical usefulness in improving the diagnostic value of US for detection of hepatic steatosis; further studies are needed to validate the UDFF performance in larger and longitudinal cohorts of patients.
